# Repair of astrocytes, blood vessels, and myelin in the injured brain: possible roles of blood monocytes

**DOI:** 10.1186/1756-6606-6-28

**Published:** 2013-06-10

**Authors:** Hey-Kyeong Jeong, Kyung-min Ji, Jun Kim, Ilo Jou, Eun-Hye Joe

**Affiliations:** 1 Neuroscience Graduate Program, Department of Biomedical Sciences, Ajou University School of Medicine, Suwon, Kyunggi-do 443-721, Korea; 2 Department of Pharmacology, Ajou University School of Medicine, Suwon, Kyunggi-do 443-721, Korea; 3 National Research Lab of Brain inflammation, Ajou University School of Medicine, Suwon, Kyunggi-do 443-721, Korea; 4 Chronic Inflammatory Disease Research Center, Ajou University School of Medicine, Suwon, Kyunggi-do 443-721, Korea

**Keywords:** Brain inflammation, Repair, Brain microenvironment

## Abstract

Inflammation in injured tissue has both repair functions and cytotoxic consequences. However, the issue of whether brain inflammation has a repair function has received little attention. Previously, we demonstrated monocyte infiltration and death of neurons and resident microglia in LPS-injected brains (*Glia*. 2007. 55:1577; *Glia*. 2008. 56:1039). Here, we found that astrocytes, oligodendrocytes, myelin, and endothelial cells disappeared in the damage core within 1–3 d and then re-appeared at 7–14 d, providing evidence of repair of the brain microenvironment. Since round Iba-1^+^/CD45^+^ monocytes infiltrated before the repair, we examined whether these cells were involved in the repair process. Analysis of mRNA expression profiles showed significant upregulation of repair/resolution-related genes, whereas proinflammatory-related genes were barely detectable at 3 d, a time when monocytes filled injury sites. Moreover, Iba-1^+^/CD45^+^ cells highly expressed phagocytic activity markers (e.g., the mannose receptors, CD68 and LAMP2), but not proinflammatory mediators (e.g., iNOS and IL1β). In addition, the distribution of round Iba-1^+^/CD45^+^ cells was spatially and temporally correlated with astrocyte recovery. We further found that monocytes in culture attracted astrocytes by releasing soluble factor(s). Together, these results suggest that brain inflammation mediated by monocytes functions to repair the microenvironment of the injured brain.

## Background

Brain inflammation accompanied by brain injury has been a focus of research efforts because of its possible roles in the onset and progression of a number of neurodegenerative diseases. However, most brain inflammation studies have focused on neurons, and little information on how brain inflammation affects other brain cells, including astrocytes and oligodendrocytes, is available. Astrocytes function to maintain the homeostasis of the brain microenvironment by taking up potassium, glutamate, and water from the extracellular space, and supplying nutrients and growth factors to neurons (for review, see [1]). Oligodendrocytes myelinate axons, allowing for rapid propagation of action potentials to axon terminals. Therefore, it is important to know how these cells respond to brain injury.

Studies of systemic inflammation showed that inflammation has dual functions: a cytotoxic function to kill infected microbes and a repair function to regenerate damaged tissues [2,3]. In the presence of proinflammatory stimulators such as IFN-γ, monocytes/macrophages are classically activated and protect the tissue from infection by producing cytotoxic inflammatory molecules [4,5]. On the other hand, in the presence of IL-4 and IL-13, monocytes/macrophages are alternatively activated, and produce several molecules that are involved in anti-inflammation and repair/regeneration [6-9]. Therefore, in myocardial injury, monocytes/macrophages rapidly remove cell debris and lead to myofibroblast infiltration and collagen deposition through production of high levels of TGF-β and VEGF-A [8]. In injured skin, macrophage depletion causes delays in healing processes [10,11].

Previously, we reported that in lipopolysaccharide (LPS)- or ATP-injected brain and contusion-induced spinal cord, resident microglia as well as neurons die in the damage core and monocytes appear and fill the damage core [12-15]. In the ischemic brain, infiltration of blood monocytes has been reported [16,17]. However, it is not clear how monocytes contribute to brain inflammation and what occurs after monocytes infiltrate into the injured brain.

In the present study, we examined how astrocytes, oligodendrocytes, and endothelial cells respond to damage in the LPS-injected substantia nigra (SN), a Parkinson’s disease-related brain area where LPS induces significant damage [13]. We also examined the roles of infiltrating monocytes in the injured brain. The results of this study showed that damage to astrocytes, oligodendrocytes, and endothelial cells peaked at approximately 3 d. However, these cells recovered 7–14 d after infiltration from the blood of round Iba-1^+^/CD45^+^ monocytes. Importantly, these monocytes expressed repair/regeneration-related genes, suggesting that they may function to repair the damaged brain.

## Results

### Recovery of the damaged microenvironment in the injured brain

Since most studies of brain injury have focused on neuronal damage, knowledge is limited on how other brain cells behave in the injured brain. In the present study, we first examined the time-dependent responses of brain cells, including astrocytes, endothelial cells, oligodendrocytes (by examining myelin) and inflammatory cells, in the injured brain. To achieve this, LPS (5 μg in 2 μl PBS) was injected into the substantia nigra pars compacta (SNpc); this region was chosen based on our previous observation that LPS induces significant damage to the SNpc but not to the cortex [13].

**Figure 1:**
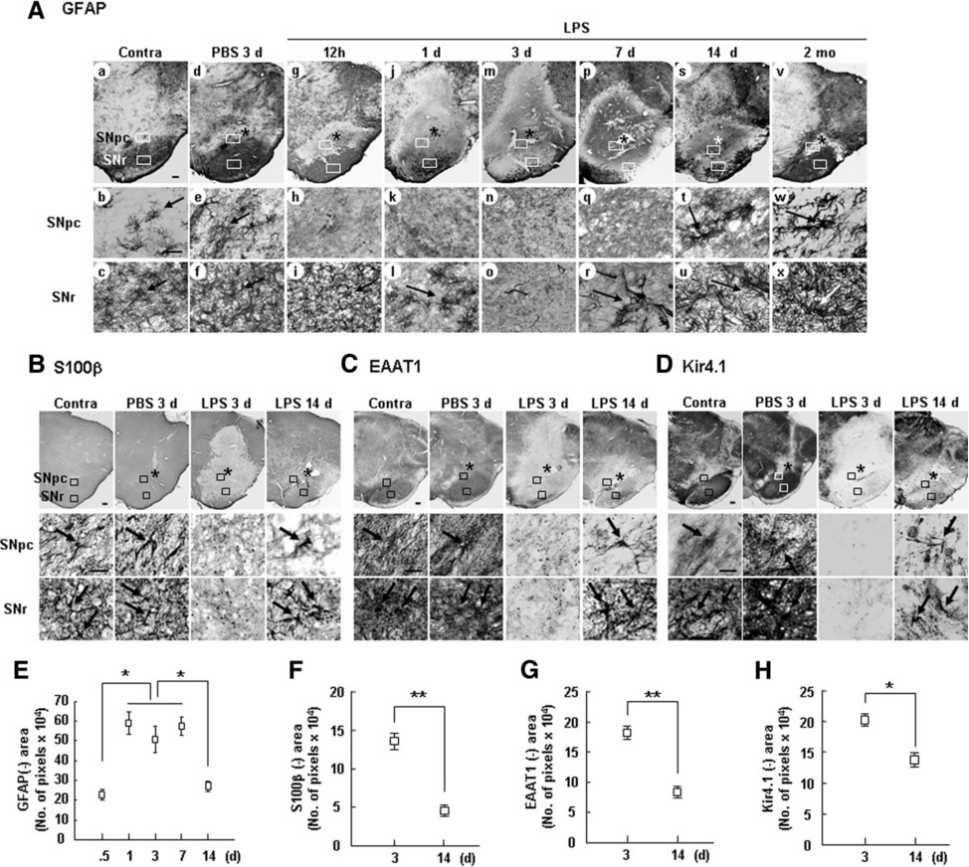
**Impaired astrocytes were subsequently recovered in the LPS-injected SNpc.** (**A**-**D**) LPS (5 μg/2 μl) was injected into the SNpc. Sections obtained at the indicated times after LPS injection were labeled with antibodies specific for GFAP (**A**), S100β (**B**), EAAT1 (**C**), and Kir 4.1 (**D**), and visualized with peroxidase-conjugated secondary antibodies. Lower panels (SNpc and SNr) are higher magnification images of boxed areas (SNpc and SNr) in upper panels. Arrows indicate GFAP^+^ (**A**), S100β^+^ (**B**) EAAT1^+^ (**C**) and Kir4.1^+^ (**D**) cells. Contralateral (Contra) sides of LPS-injected animals and PBS-injected animals were used as controls. Asterisks (*) indicate injection sites. Scale bars, 1 mm (upper panels in **A**), 200 μm (upper panels in **C**-**E**), 50 μm (lower panels in **A**), 20 μm (lower panels in **C**-**E**). (**E**-**H**) GFAP-, S100β-, EAAT1-, and Kir 4.1-negative areas in every sixth brain section were quantified using Axiovision image analysis software, as described in “Materials and Methods”. Values are means ± SEMs of at least three samples (*p < 0.01; **p < 0.001; Student’s *t*-test). Three or more animals were used for each time point. All data presented in this study are representative of at least three independent experiments unless indicated.

In the intact brain, the density of GFAP^+^ astrocytes (arrows in Figure 1Ab) differs among regions; within the SN, it is low in the SNpc and high in the substantia nigra pars reticulata (SNr) (Figure 1Aa-c). LPS injection into the SNpc induced the death of astrocytes within 12 h (Figure 1Ag, h), as we and others have previously reported [13,18]. At 1–3 d, GFAP^+^ astrocyte-empty areas were significantly expanded (Figure 1Aj-o). In the SNr, the number of astrocytes was reduced on day 1 (Figure 1Al), and these cells almost disappeared by day 3 (Figure 1Ao). In response to PBS-injection, astrocytes became activated − the number of astrocyte processes increased and the processes became longer and thicker (arrow in Figure 1Ae) − but death did not occur (Figure 1Ad-f). Interestingly, however, the impaired astrocytes recovered between 7 and 14 d, although they exhibited a highly activated morphology (Figure 1Ar, t, u). At 2 mo, astrocytes filled the damaged areas almost completely (Figure 1Av), although these cells were still activated (arrows in Figure 1Aw, x).

The disappearance and reappearance of GFAP^+^ astrocytes were not merely due to reduced GFAP immunoreactivity since other markers of astrocytes in addition to GFAP, including S100β (Ca^2+^-binding protein), EAAT1 (GLAST, excitatory amino acid transporter 1) and Kir4.1 (potassium channel), also disappeared at 3 d (Figure 1B-D) and reappeared at 14 d (arrows in Figure 1B-D). The areas in which each marker was absent were measured and plotted (Figure 1E-H). Using Nissl staining, we verified the death of astrocytes at 1 d (Additional file 1: Figure S1).

**Figure 2:**
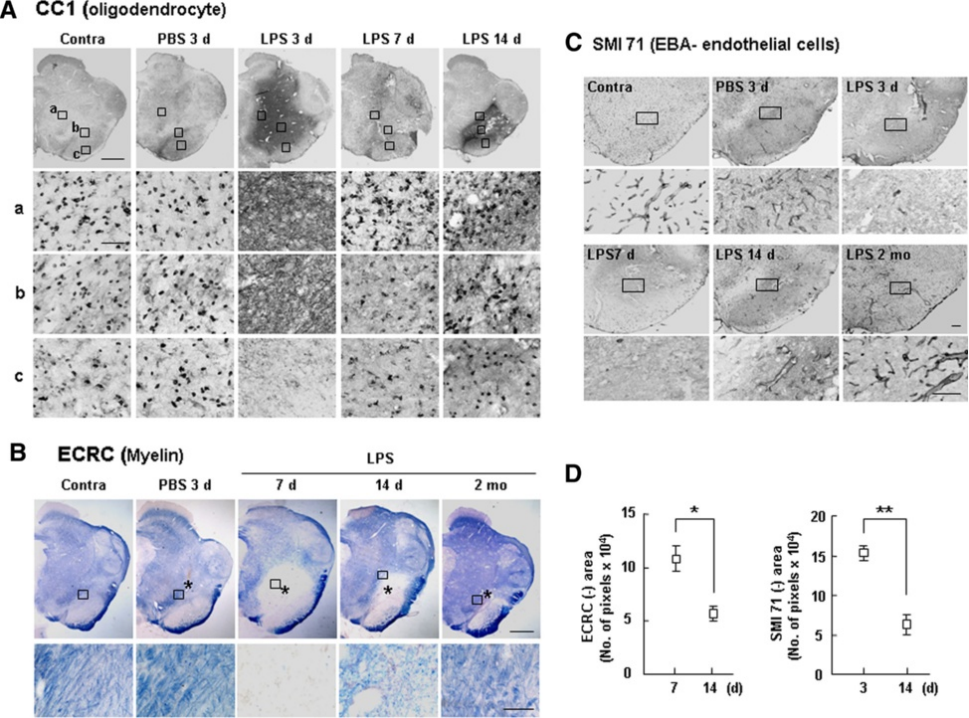
**Recovery of impaired oligodendrocytes, myelin, and endothelial cells in LPS-injected SNpc.** (**A**, **C**) At the indicated times after LPS injection, sections were labeled with antibodies specific for markers of oligodendrocytes (CC1) and endothelial cells (SMI 71). **(B)** For myelin staining, sections were stained with ECRC. (**A**-**C**) Lower panels are higher magnification images of boxed areas in upper panels. Contralateral (Contra) sides of LPS-injected animals and PBS-injected animals were used as controls. **(D)** ECRC- and SMI 71-negative areas were quantified at the indicated times. Values are means ± SEMs of more than three samples (*p < 0.01; **p < 0.001; Student’s *t*-test). Asterisks (*) indicate drug injection sites. Scale bars, 1 mm (upper panels in **A**, **B**), 200 μm (upper panels in **C**), 100 μm (lower panels in **B**, **C**), and 50 μm (lower panels in **A**).

**Figure 3:**
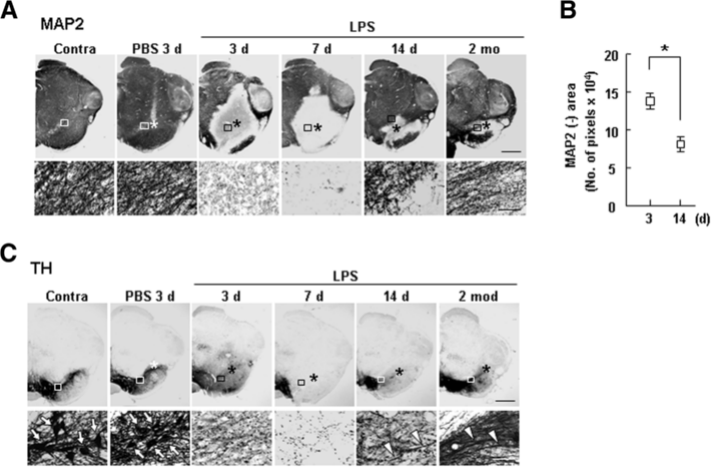
**Recovery of impaired neurites in LPS-injected SNpc.** (**A**, **C**) At the indicated times after LPS injection, sections were labeled with antibodies specific for markers of MAP2 (**A**) and tyrosine hydroxylase (TH) (**C**). Lower panels are higher magnification images of boxed areas in upper panels. Arrows in lower panels in (**C**) indicate TH^+^ cell bodies. **(B)** MAP2-negative areas at 3 and 14 d after LPS injection were quantified. Values are means ± SEMs of more than three samples (*p < 0.01; Student’s *t*-test). Asterisks (*) indicate injection sites. **(C)** In contralateral (contra) sides and PBS-injected brains, TH^+^ dopaminergic neuronal cell bodies were detectable (white arrows), but TH^+^ dopaminergic neuronal cell bodies and processes were injured at 3 d after LPS injection. At 14 d and 2 mo, only processes reappeared (white arrowheads). Scale bars, 1 mm (upper panels in **A**, **C**), and 100 μm (lower panels in **A**, **C**).

We next examined oligodendrocytes and myelin. CC-1^+^ oligodendrocytes were injured at 3 d and gradually recovered beginning on day 7 (Figure 2A). Similarly, the decrease in Eriochrome Cyanine RC (ECRC)-stained myelin also reached a peak at 3–7 d, but gradually recovered at 14 d and 2 mo (Figure 2B, D). Blood vessels with SMI 71^+^ endothelial cells disappeared in the injection core at 3 d after LPS injection, but reappeared at 14 d, and seemed to be almost intact at 2 mo (Figure 2C, D). Interestingly, the neuron-damage area detected by MAP2^+^ dendrites reached a peak at 3 d and was maintained and/or slightly decreased at 7 d before decreasing gradually at later time points (14 d and 2 mo) (Figure 3A, B). TH-positivity also showed a similar pattern. In the contralateral (contra) side and in PBS-injected controls, TH^+^ dopaminergic neuronal cell bodies were detectable (Figure 3C, white arrows), but both TH^+^ dopaminergic neuronal cell bodies and processes were injured at 3 d after LPS injection. Notably, however, only processes (white arrowheads), but not cell bodies, reappeared at 14 d and 2 mo (Figure 3C). These results suggest that brain microenvironmental damage (damage to astrocytes, oligodendrocytes, myelin, blood vessels, and neurites) actively recovers in the injured brain.

### Astrocytes and oligodendrocytes proliferate and migrate toward the damage

**Figure 4:**
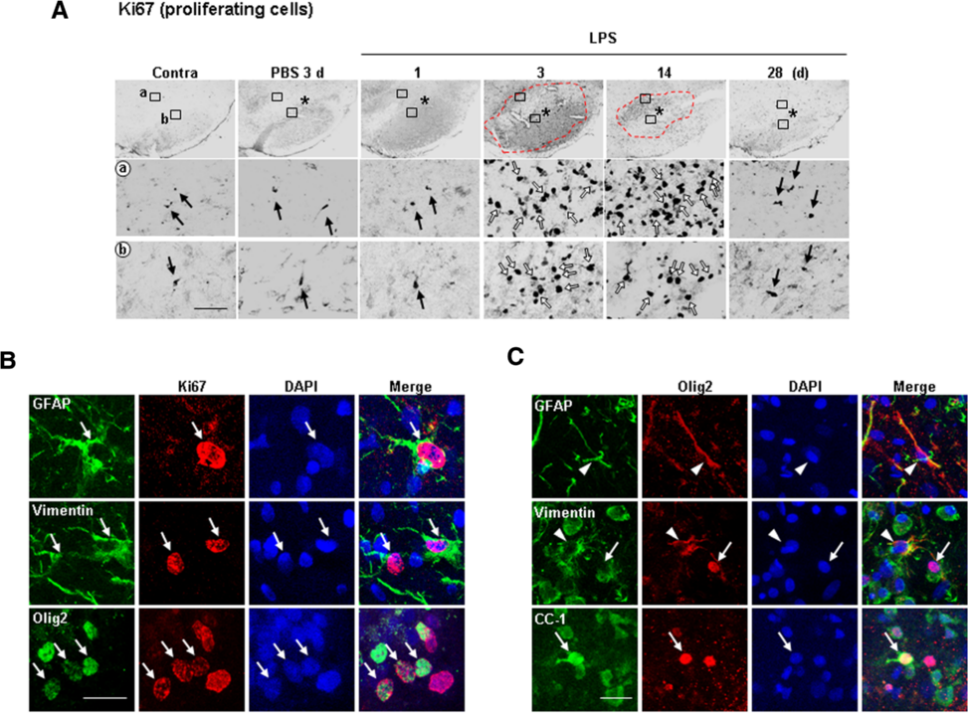
**Ki-67 immunoreactivity was found in astrocytes and oligodendrocytes in LPS-injected SNpc.** (**A**) Brain sections were prepared at the indicated times after LPS-, and PBS-injection and stained with antibodies specific for Ki-67. Lower panels are higher magnification of boxed areas in upper panels. In intact contralateral (contra) sides and sections from PBS-injected brains, some Ki67^+^ cells were detectable (black arrows). Dotted lines at 3 and 14 d show areas where the number of Ki67^+^ cells dramatically increased (whiter arrows). Asterisks (*) indicate injection sites. **(B)** Sections obtained at 3 d were double-labeled with combinations of Ki-67/GFAP, Ki-67/Vimentin, and Ki67/Olig2 antibodies. Nuclei were labeled with DAPI. Arrows indicate cells co-labeled with Ki67 and each cellular marker. **(C)** Sections obtained at 7 d were double-labeled with combinations of Olig2/Vimentin and Olig2/CC-1 antibodies. Arrowheads and arrows indicate cells where Olig2 was found in the cytosol and nuclei, respectively. Scale bars, 200 μm (upper panels in **A**), 50 μm (lower panels in **A**), and 20 μm (**B**, **C**).

We further examined whether the impaired astrocytes could be recovered by cell proliferation. Cells expressing Ki-67 (a cell cycle protein used as a proliferation marker) significantly increased from 3 d (Figure 4A), while Ki-67^+^ cells were barely detectable in intact SNpc and at 1 d after LPS injection (Figure 4A). In double-labeling experiments, proliferating Ki67^+^ cells were merged with GFAP^+^ and/or vimentin^+^ astrocytes (Figure 4B). In addition, Ki67 immunoreactivity was found in Olig2^+^ cells, which are considered progenitor cells of oligodendrocytes and astrocytes, as well as in reactive astrocytes [19,20] (Figure 4B). Interestingly, Olig2 was detected in either the nucleus (arrows) or cytosol (arrowheads) of GFAP^+^ or vimentin^+^ astrocytes and CC-1^+^ oligodendrocytes 7 d after LPS injection (Figure 4C). These findings suggest that astrocytes and oligodendrocytes proliferate and fill in astrocyte- and oligodendrocyte-deficient regions in the LPS-injected SNpc.

### Possible involvement of brain inflammation in repair of damaged microenvironment of the brain

**Figure 5:**
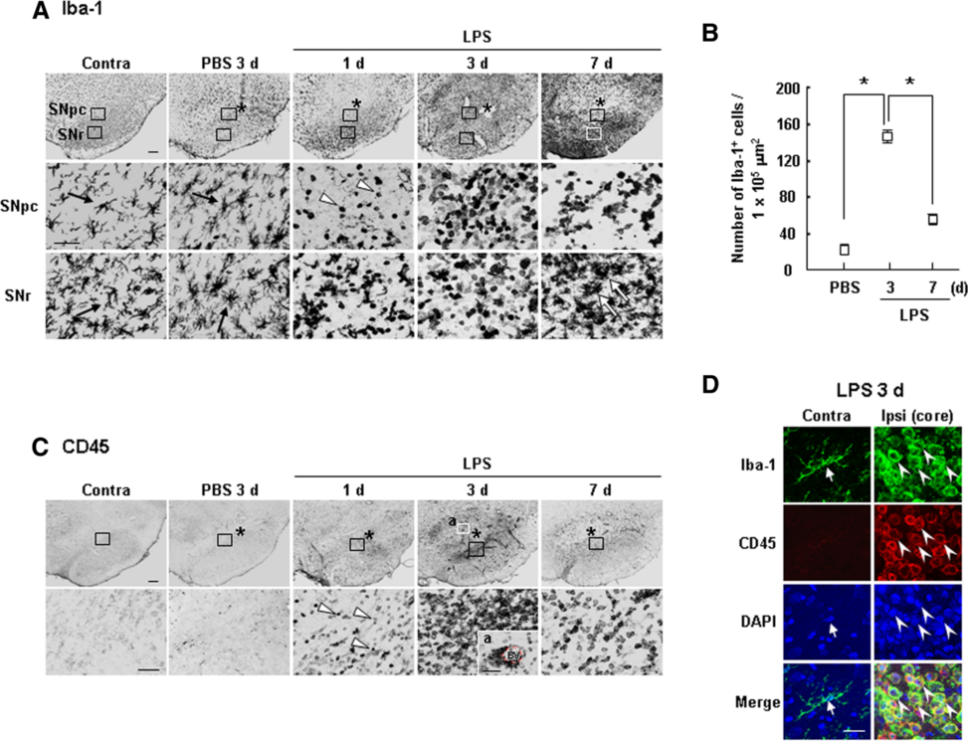
**Appearance of Iba-1**^**+**^**/CD45**^**+ **^**round cells in the LPS-injected SNpc.** (**A**, **C**, **D**) Serial sections were obtained at the indicated times after LPS injection and labeled with Iba-1 (**A**), CD45 (**C**), and Iba-1/CD45 (**D**) antibodies. (**A**, **C**) Lower panels are higher magnification images of the boxed areas in the upper panels. **(A)** In intact contralateral (Contra) sides and PBS-injected brains, Iba-1^+^ cells were ramified (black arrows). In LPS-injected brain, round Iba-1^+^ cells were detectable at 1 d (white arrowheads), and the number of these cells increased at 3 d but decreased at 7 d. In SNr areas, Iba-1^+^ cells became ramified and highly expressed Iba-1 at 7 d (white arrows). **(B)** Iba-1^+^ cells at the indicated times after LPS injection were quantified. Values are means ± SEMs of more than three samples (*p < 0.001; Student’s *t*-test). **(C)** CD45^+^ cells were detectable at 1 d (white arrowheads), and the number of these cells increased at 3 d but decreased at 7 d. Some round CD45^+^ cells were found around blood vessels (BV) (inset ‘a’ in 3 d). **(D)** Brain sections obtained at 3 d were double-labeled with Iba-1 and CD45 antibodies, and visualized with Alexa Fluor 488- and 555-conjugated antibodies, respectively. Asterisks (*) indicate drug injection sites. Scale bars, 200 μm (upper panels in **A**, **C**), 50 μm (lower panels and the inset in **A**, **C**), and 20 μm (**D**).

Because an important role of inflammation is to repair damaged tissue [8,10] we examined whether brain inflammation could contribute to the repair of damage to the brain microenvironment. Previously, we reported that ramified Iba-1^+^ resident microglia died in injured brain and spinal cord, and that round Iba-1^+^ monocytes infiltrated into these tissues [12-15]. In this study, we found that in LPS-injected brain, the number of round Iba-1^+^ cells was markedly increased at 3 d, but subsequently decreased between 3 and 7 d (Figure 5A, B). In the SNpc, the decrease in the number of round Iba-1^+^ cells at 7 d appeared to be due to the death of a portion of these cells, since some round Iba-1^+^ cells were positive in TUNEL assays (lower panels ‘SNpc’ in Figure 5A, Additional file 2: Figure S2). In SNr areas, Iba-1^+^ cells became ramified and highly expressed Iba-1 at 7 d (lower panels ‘SNr’ in Figure 5A, white arrows). In the intact brain, the density of Iba-1^+^ microglia was low in the SNpc and high in the SNr, as shown in the contralateral (Contra) side. We speculate that, during repair processes, more monocytes survive in the SNr and become resident microglia. Additionally, round Iba-1^+^ cells were not detectable in the PBS-injected brain (Figure 5A). Round CD45^+^ cells also appeared approximately 1 d after LPS injection whereas they were not detectable in intact and PBS-injected brains (Figure 5C). Particularly, these CD45^+^ cells were found around blood vessels (BV) with high densities (inset in Figure 5C lower panel ‘a’). The number of CD45^+^ cells and their recruited area also reached a maximum at 3 d, and then progressively decreased through to 7 d (Figure 5C). Double-labeling experiments additionally showed CD45 immunoreactivity in round Iba-1^+^ cells in the ipsilateral sides at 3 d (arrowheads in Figure 5D) but not in ramified Iba-1^+^ cells in the contralateral sides (arrows in Figure 5D). It has been reported that monocytes highly express Iba-1 and CD45, whereas resident microglia express Iba-1 but weakly and barely express CD45 [12,14,21-24] (Table 1). Previously, we reported that labeled monocytes transplanted into the tail vein were found in the damage core in LPS-injected brains [12]. Therefore, based on these lines of evidence, we considered the round Iba-1^+^ and/or CD45^+^ cells as monocytes.

**Table 1:** Primary antibodies used to detect microglia/monocytes

**Name**	**Detectable cell types**	**References**
CD11b	Brain microglia	[21]
	Monocytes/macrophages	
CD45	Monocytes (CD45^high^)	[14,22,24]
	Microglia (CD45^low^)	
Iba-1	Brain microglia	[12,22,23]
	Monocytes/macrophages	

**Figure 6:**
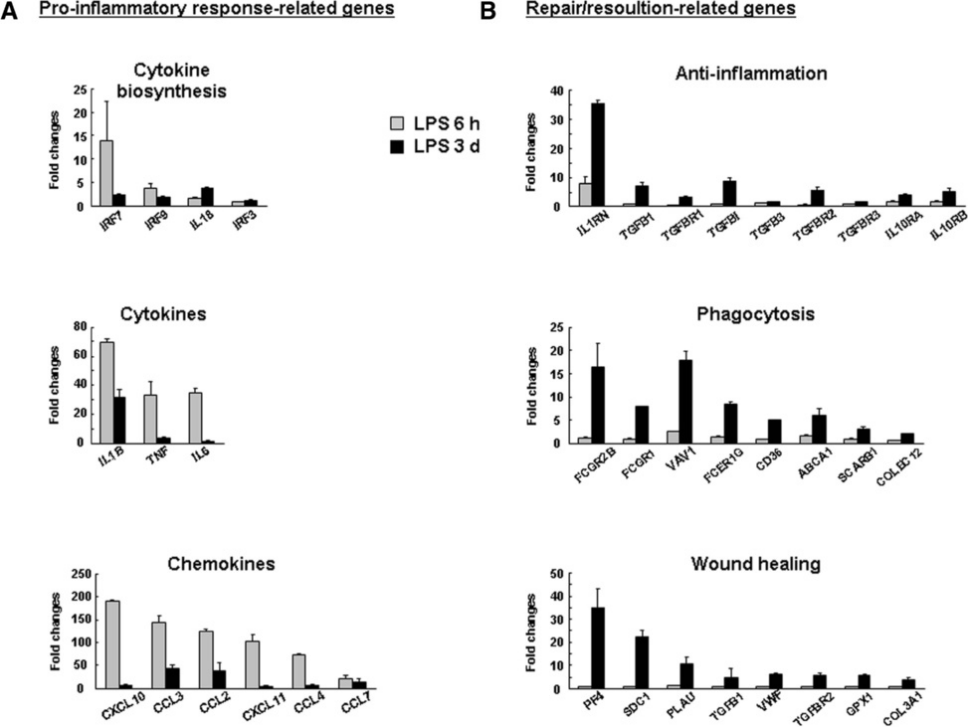
**Microarray analysis of mRNA expression in the LPS-injected SNpc.** mRNA expression profiles were compared in intact, and 3 h and 3 d after LPS-injected brain. For each microarray sample, brain tissue blocks (2 × 2 × 2 mm) were obtained from four animals, and total RNA was extracted as described in “Materials and Methods”. Relative mRNA levels of proinflammatory response-related genes (**A**) and repair/resolution-related genes (**B**) at 6 h and 3 d after LPS injection are shown as fold-changes normalized to that in the intact SNpc. Data shown are representative of two independent experiments.

Next, using a microarray, we examined mRNA expression patterns at times before (6 h) and after (3 d) infiltration of monocytes, respectively. At 6 h, mRNA expression of cytokines (IL1B, TNF, IL6), chemokines (CCL2, 3, 4, 7, CXCL 10 and 11), and transcription factors that regulate biosynthesis of cytokines and chemokines (IRF3, 7, and 9) significantly increased in the LPS-injected brain, but barely increased at 3 d (Figure 6A). In contrast, expression of genes associated with anti-inflammation (IL1RN, TGFB1, TGFBI, TGFBR1, IL10RA, IL10RB), phagocytosis (COLEC12, CD36, SCARB1, FCGR1), and wound healing (TGFB1, TGFBR2, PF4, VWF, COL3A1) were significantly increased at 3 d, but were barely elevated or decreased at 6 h after LPS injection (Figure 6B).

**Figure 7:**
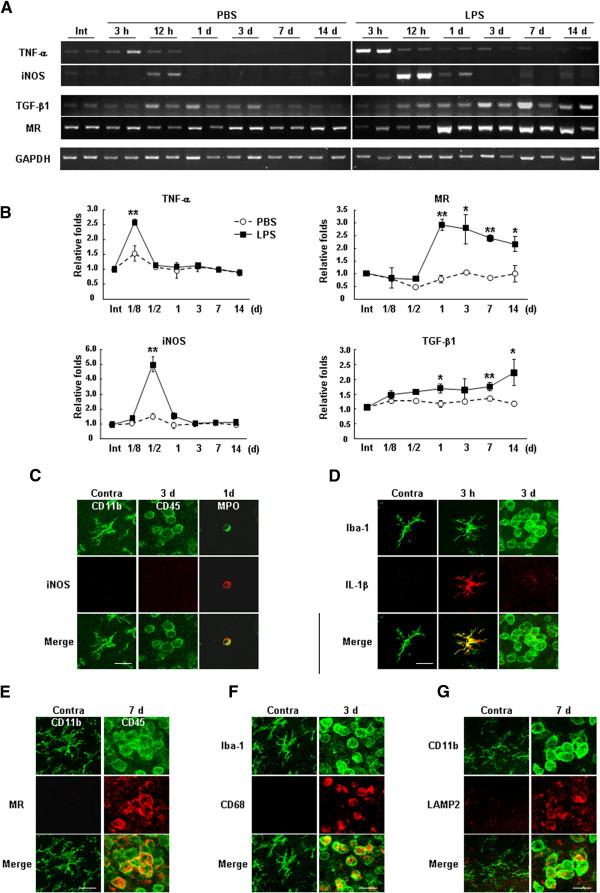
**Iba-1**^**+**^**/CD45**^**+ **^**round cells in LPS-injected SNpc were alternatively activated.** (**A**, **B**) At the indicated times after LPS or PBS injection, brain tissue blocks (2 × 2 × 2 mm) were obtained, and the levels of TNF-α, iNOS, TGF-β1, and MR mRNA were analyzed by RT-PCR (**A**). Band intensities in (**A**) were analyzed and plotted (**B**). Intact (Int) SNpc was used as a control. (**C**-**G**) Sections were obtained at the indicated times after LPS injection and labeled with combinations of markers of microglia/monocytes (CD11b, CD45, Iba-1), neutrophils (MPO, myeloperoxidase), proinflammatory mediators (iNOS and IL-1beta), and alternative activation and phagocytosis (MR, CD68, and LAMP2). iNOS was detected in neutrophils and IL-1beta in ramified cells, respectively. All of MR, CD68, and LAMP2 were detected in round cells but not in ramified cells. Values in (**B**) are means ± SEMs of more than three samples (*p < 0.05; **, p < 0.001 for PBS vs. LPS; Student’s *t*-test). Data shown in (**A** and **C**-**G**) are representative of more than three samples. Intact SN region (Int) and PBS injection were used as controls. Scale bars, 20 μm (**C**-**G**).

We further analyzed gene expression by RT-PCR, focusing on the expression of two prominent inflammatory mediators, TNF-α and iNOS, and two markers of repair/resolution-related genes, mannose receptor (MR) and TGF-β1 [7]. In the LPS-injected brain, expression of TNF-α and iNOS mRNA increased at times before infiltration of monocytes (3 and 12 h) compared with the PBS-injected brain, but was barely detectable at 1 d and thereafter (Figure 7A, B). On the other hand, mRNA expression of MR significantly increased from 1 d, and was maintained at an elevated level for up to 14 d (Figure 7A, B). TGF-β1 mRNA levels slightly increased in LPS-injected brain at 12 h-1 d after LPS injection, and remained elevated for up to 14 d (Figure 7A, B). Taken together, these results suggest that gene expression patterns in LPS-injected SN after monocyte infiltration show repair/resolution-related function rather than proinflammatory and/or neurotoxic function.

Next, we used double-immunolabeling to examine whether monocytes expressed proinflammatory genes and repair/resolution-related genes in the injured brain. Depending on the sources of antibodies used to examine expression of proteins, microglia and/or monocytes were identified by staining for Iba-1, CD11b, or CD45 in these cells (Table 1). Expression of iNOS, a major inflammatory mediator, was detected in myeloperoxidase (MPO)^+^ neutrophils at 12 h to 1 d after LPS injection, but not in CD11b^+^ or CD45^+^ cells (Figure 7C), as we previously reported [12]. IL-1β expression was detected in ramified Iba-1^+^ cells at 3 h (Figure 7D). However, round CD45^+^ and Iba-1^+^ cells expressed neither iNOS nor IL-1β at 3–7 d (Figure 7D). Interestingly, most round CD45^+^ cells expressed mannose receptor (MR), which is known to play important roles in endocytosis/phagocytosis [25,26] (Figure 7E). In addition, round but not ramified Iba-1^+^ cells highly expressed CD68, an indicator of lysosomal enzyme activity that is considered a marker of phagocytic activity [27] (Figure 7F). Round CD11b^+^ cells also highly expressed LAMP2 (Figure 7G), a lysosomal protein that participates in the fusion of lysosomes and phagosomes [28]. Since these monocytes have strong phagocytic activity, they may scavenge damaged cells and debris in the injured brain. These results suggest that monocytes may contribute to the recovery of impaired astrocytes in LPS-injected SNpc.

### Possible spatial and temporal correlation between astrocytes and monocytes cells in the injured brain

**Figure 8:**
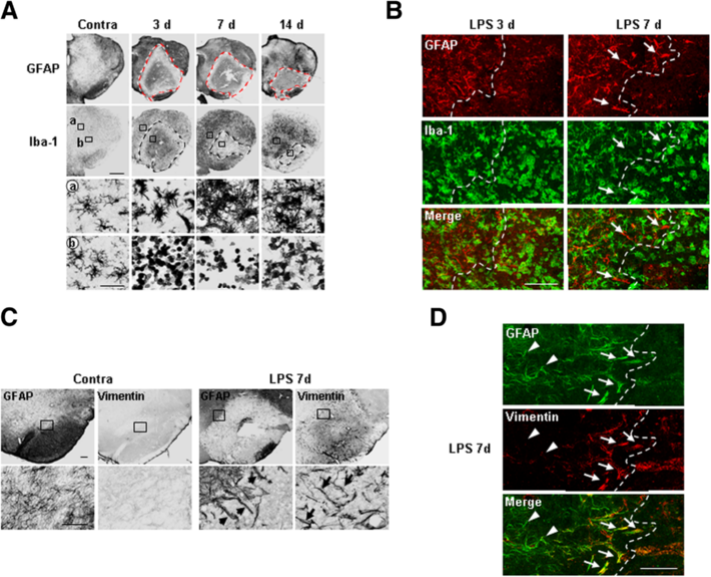
**Possible spatial and temporal correlation between astrocytes and round Iba-1**^**+ **^**cells in the injured brain.** Brain sections (30 μm) were obtained at the indicated times after LPS injection. (**A**, **C**) Immediately adjacent serial sections were stained with GFAP, Iba-1, and Vimentin antibodies, respectively. Lower panels are higher magnification of boxed areas in upper panels. (**B**, **D**) Sections were double-labeled with GFAP/Iba-1 (**B**) or GFAP/Vimentin (**D**). Arrows indicate astrocyte processes protruded towards monocytes (**B**, **C**). Arrows and arrowheads indicate Vimentin^+^ astrocytes near monocytes and Vimentin^-^ astrocytes far from monocytes, respectively (**D**). Dotted lines in (**A**, **B**, and **D**) indicate the borders where GFAP^+^ astrocytes were absent and/or Iba-1^+^ round monocytes were present. Data shown are representative of at least three independent experiments. In each experiment, three or more animals were used for each time point. Scale bars, 1 mm (**A**), 200 μm (upper panels in **C**), 100 μm (**B**, lower panels in **C**, and **D**), 50 μm (lower panels in **A**).

Since recovery of the damaged microenvironment was detected in the damaged core filled with monocytes, we further examined whether correlation between repair of astrocytes and infiltration of monocytes in the injured brain. In results, we observed a correlation between the distribution of round monocytes and astrocytes: astrocytes expanded their territory as the area occupied by round Iba-1^+^/CD45^+^ cells decreased in a time-dependent manner (dotted lines in Figure 8A). In addition, astrocytes protruded their processes toward the damage core filled with monocytes at 7 d (arrows in Figure 8B, C). Furthermore, Vimentin, which positively regulates migration of cells including astrocytes [29,30], was detectable in the protruded astrocyte processes near the damage core (arrows in Figure 8C, D), but not in astrocytes far from monocytes (arrowheads in Figure 8D).

**Figure 9:**
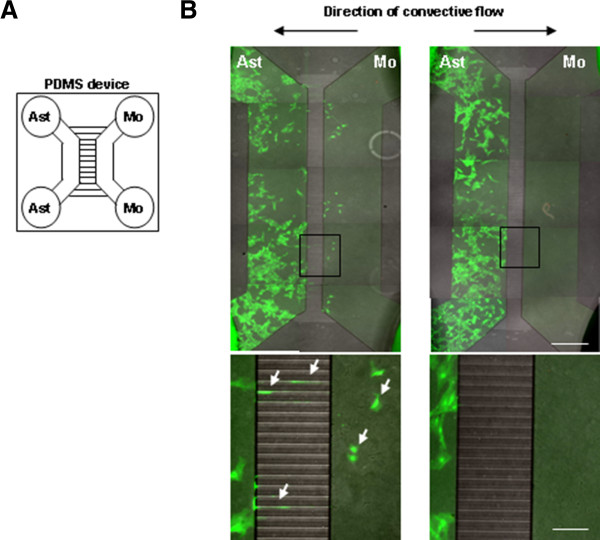
**Monocytes facilitate astrocyte migration.** Astrocytes and monocytes were co-cultured in the each compartment of PDMS devices (**A**) as described in “Materials and Methods”. (**B**) At 7 d after co-culture, astrocytes were visualized with calcein AM. Convective flow was generated by the difference in media volumes (250 vs. 300 μl) in the compartments in PDMS. The volume difference was maintained to day 7. Astrocytes migrated toward monocytes through the groove only when the convective flow was from monocytes to astrocytes (white arrows in **B**, left panel). The lower panels in (**B**) represent higher magnification of the indicated areas in the upper panels. Bars 200 μm (**B** upper panel); 50 μm (**B** lower panel).

Next, we examined whether monocytes could encourage migration of astrocytes in culture. We measured migration ability of astrocytes using the PDMS device (Figure 9A). Astrocytes (Ast) and monocytes (Mo) were plated in each side of the groove. At 7 d after plating, astrocytes migrated toward monocytes when the direction of convective flow was from monocytes to astrocytes (Figure 9B left panel) but not when the direction was from astrocytes to monocytes (Figure 9B right panel). In addition, astrocytes did not move toward dead monocytes or toward media alone (data not shown). Taken together, these results suggest that monocytes in brain inflammation may contribute to recovery of damaged astrocytes by promoting astrocyte migration.

## Discussion

The results in this study showed that 1) astrocytes, oligodendrocytes, myelin, and endothelial cells recovered in the injured brain; 2) the recovery of these cells and myelin occurred after infiltration of Iba-1^+^/CD45^+^ monocytes into injury sites; 3) Iba-1^+^/CD45^+^ monocytes expressed repair-related inflammatory mediators, but not cytotoxic proinflammatory mediators; and 4) *in vitro*, monocytes secreted soluble factor(s) that recruited astrocytes toward them. On the basis of these findings, we suggest that monocytes may function to repair the injured brain.

Many studies on cultured microglia have reported that brain inflammation is neurotoxic. However, brain inflammation *in vivo* is quite different from the inflammation associated with microglia in culture [12-15,31]. Simply, in the brain, several types of cells including microglia, astrocytes, neurons, and blood inflammatory cells, both positively and negatively contribute to inflammation. Microglia continuously survey brain damage, isolate damaged areas [14,32], and recruit monocytes [33]. Astrocytes and/or neurons inhibit microglial activation [31,34-37] and recruit monocytes [38]. Neutrophils produce cytotoxic inflammatory mediators in the brain [12,17]. However, the roles of monocytes in the brain have not been clearly defined. Since monocytes/macrophages can be differentially activated (cytotoxic/classical vs. repair/alternative activation) depending on the stimuli, their roles also differ according to the activation conditions [7,9]. The results presented here showed that monocytes in the LPS-injected brain exhibited repair-promoting rather than neurotoxic phenotypes. In microarray, RT-PCR, and immunohistochemistry analyses, repair/resolution-related genes/proteins such as phagocytic activation markers were highly expressed in the LPS-injected brain during the period when monocytes appeared, whereas cytotoxic proinflammatory mediators were barely expressed (Figures 6, 7). Phagocytosis is an important process for the repair/regeneration of damaged tissue because damaged cells and debris may act as detrimental factors that lead to further injury or hinder regeneration [10]. In a multiple sclerosis animal model, stimulation of phagocytosis was shown to increase clearance of tissue debris, limit further destruction, and facilitate repair [39].

The question arising from the above considerations is how monocytes have a repair function instead of a neurotoxic function in the LPS-injected brain. There is a remote possibility that monocytes are activated by LPS since cerebrospinal fluid is exchanged about 11 times daily to maintain homeostasis of the brain in the adult rat brain [40]. Monocytes may be alternatively activated by phagocytosis of apoptotic cells. It has been reported that phagocytosis alters the phenotypes of monocytes from a proinflammatory to an anti-inflammatory phenotype [41]. Another possibility is that neutrophils that enter the brain prior to monocytes may induce alternative activation because neutrophils express IL-4, a strong inducer of alternative activation [6,12,42].

Next, we examined the issue of how astrocytes, oligodendrocytes, myelin, endothelial cells, and neurites reappeared in damaged areas. We detected Ki-67^+^ proliferating cells in the injured area, and these cells were merged with GFAP, Vimentin, and Olig2 (Figure 4), suggesting that astrocytes and oligodendrocytes proliferate and fill the damaged area. Interestingly, Olig2 immunoreactivity was located in the cytosol of GFAP^+^/Vimentin^+^ astrocytes and in the nuclei of CC-1^+^ oligodendrocytes (Figure 4C). It has been reported that Olig2 is expressed in progenitor cells of oligodendrocytes and astrocytes, as well as in reactive astrocytes in the injured brain [19,20]. As we also showed in Figure 4C, it has been reported that Olig2 is located in the cytosol and nuclei of cells that are destined to become astrocytes and oligodendrocytes, respectively [43,44]. Therefore, newly generated Ki67^+^/Olig2^+^ progenitors may replenish astrocytes and oligodendrocytes in the injured brain. It has been reported that monocytes express several chemokines, including IL-8, IP-10, CCL1, CCL2 (MCP-1), and CCL4 [45,46]. Monocytes also produce platelet-derived growth factor (PDGF), transforming growth factor beta (TGF-β), and hepatocyte growth factor (HGF) [47-49]. Therefore, monocytes may recruit and/or promote the proliferation of astrocytes and oligodendrocytes, and induce neurite outgrowth. These findings suggest that brain inflammation plays a role in repairing damaged brain tissue. Thus, we speculate that impairment of the repair function of inflammation due to aging or genetic mutations may result in delayed recovery from damage and neurodegeneration. In our microarray analysis, mRNA expression of several genes related to cell proliferation/migration and neurogenesis was upregulated at around the times when monocytes appeared in the brain (data not shown). Furthermore, we observed that, in culture, monocytes promote astrocyte migration toward them (Figure 9), suggesting that monocytes in the LPS-injected brain may attract astrocytes toward damaged areas for recovery. We expected that the recovery of oligodendrocytes might be similar to that of GFAP^+^ astrocytes, since they come to occupy the same microenvironment. In addition, astrocytes may contribute to the recovery of endothelial cells and neurite outgrowths since astrocytes also express neurotrophic factors and growth factors [50,51]. Taken together, these observations suggest that monocytes may assist in regeneration of the brain microenvironment in the injured brain.

## Conclusion

In the present study, impaired astrocytes, endothelial cells, and neurites were recovered, and myelination occurred, after monocytes had filled the lesion sites. We thus speculate that brain inflammation mediated by monocytes serves to repair damage in the injured brain. Our results highlight the physiological importance of brain inflammation in enhancing beneficial effects while minimizing harm.

## Materials and methods

### Ethics statement

All experiments were performed in accordance with the approved animal protocols and guidelines established by the Ajou University School of Medicine Ethics Review Committee, and all animal work was approved by the Ethical Committee for Animal Research of Ajou University (Amc-28).

### Stereotaxic surgery and drug injection

Male Sprague–Dawley rats (230–250 g, 7 weeks old) were anesthetized by ketamine (40–80 mg/kg) and xylazine (5–10 mg/kg), and positioned in a stereotaxic apparatus (David Kopf Instruments, Tujunga, CA, USA). LPS (5 μg in 2 μl sterile PBS; Sigma, St. Louis, MO, USA) was unilaterally administered into the right SNpc (AP, -5.3 mm; ML, -2.3 mm; DV, -7.6 mm from the bregma), according to the atlas of Paxinos and Watson [52]. All animals were injected using a Hamilton syringe equipped with a 30-gauge blunt needle and attached to a syringe pump (KD Scientific, New Hope, PA, USA). LPS was infused at a rate of 0.4 μl/min. After injection, the needle was held in place for an additional 5 min before removal.

### Tissue preparation

For immunostaining, rats were anesthetized and transcardially perfused with saline solution containing 0.5% sodium nitrate and heparin (10 U/ml), followed by 4% paraformaldehyde in 0.1 M phosphate buffer (pH 7.2) for tissue fixation. Brains were obtained and post-fixed overnight at 4°C in 4% paraformaldehyde. Fixed brains were stored at 4°C in a 30% sucrose solution until they sank. Six separate series of 30-μm coronal brain sections (50 μm for stereological counting) were obtained using a sliding microtome (Microm, Walldorf, Germany).

For RNA preparation, rats were anesthetized and transcardially perfused with saline solution without paraformaldehyde. Brains were obtained and sliced with a Rat Brain Slicer Matrix (1.0 mm slice intervals, RBM-4000C; ASI Instruments, Warren, MI, USA) and a razor blade. A slice that included the needle injection spot was selected, and tissue blocks (2 × 2 × 2 mm) just below the needle tip were collected and stored at −70°C until use.

### Immunohistochemistry

For 3,3′-diaminobenzidine (DAB) staining, serial sections were rinsed three times with PBS, treated with 3% H_2_O_2_ for 5 min, and rinsed with PBS containing 0.2% Triton X-100 (PBST). Non-specific binding was blocked with 1% BSA in PBST. Sections were incubated overnight at room temperature with primary antibodies (Table 2). After rinsing in PBST, sections were incubated with biotinylated secondary antibodies (Vector Laboratories, Burlingame, CA, USA) for 1 h and the avidin/biotin system (Vector Laboratories) for 1 h, and visualized using a DAB solution (0.05% DAB and 0.003% hydrogen peroxide in 0.1 M PB). Sections were then mounted on gelatin-coated slides and examined under a bright-field microscope (Olympus Optical, BX51, Tokyo, Japan). Bright-field images were obtained using PictureFrame Application 2.3 software. For immunofluorescence staining, sections were washed twice in PBS, treated with 1% BSA, and incubated with combinations of primary antibodies. For double-labeling, resident microglia and monocytes were stained for Iba-1, CD11b, or CD45 depending on the sources of antibodies against other proteins (Table 1). For visualization, Alexa Fluor 488- or Alexa Fluor 555-conjugated secondary antibodies (Invitrogen, Eugene, OR, USA) were used. DAPI (Vector Laboratories) was used to detect nuclei. Sections were analyzed under a confocal microscope (LSM 510, Carl Zeiss, Jena, Germany) with 40 × water- and 63 × oil-immersion objectives at 20°C. Images were captured using Confocal software (LSM Image Browser).

**Table 2:** Primary antibodies used in immunohistochemistry

**Antibody**	**Source**	**Dilution**	**Catalogue no.**	**Company**
APC (CC-1)	mouse	1:200	OP80	Calbiochem
CD11b	mouse	1:200	MCA275G	Serotec
CD45	mouse	1:200	MCA43R	Serotec
CD68	mouse	1:300	MCA341R	Serotec
EAAT1	rabbit	1:800	416	Abcam
GFAP	mouse	1:700	G3893	Sigma
Iba-1	rabbit	1:1000	019-19741	Wako
Ki-67	rabbit	1:200	AB9260	Chemicon
Kir4.1	rabbit	1:800	apc-035	Alomone
LAMP2	rabbit	1:200	L0668	Sigma
MAP2	mouse	1:1000	M4403	Sigma
MR	rabbit	1:200	ab64693	Abcam
Olig2	rabbit	1:200	OB-905	IBL
SMI 71	mouse	1:500	SMI-71R	Sternberger Monoclonals
S100β	rabbit	1:800	37	Swant
TH	rabbit	1:1000	P40101	Pel-Freeze
Vimentin	mouse	1:200	MAB3400	Chemicon

### Reverse transcriptase-polymerase chain reaction (RT-PCR)

Total RNA was isolated using an easy-BLUE RNA Extraction Kit (iNtRON, Sungnam, Korea), and cDNA was prepared using Reverse Transcription Master Premix (ElpisBio, Daejeon, Korea), according to the manufacturers’ instructions. Approximately 100 ng cDNA was analyzed. The specific primers for TNF-α, iNOS, TGF-β, MR, and GAPDH used in RT-PCR are shown in Table 3. RT-PCR products were verified by electrophoresis on 1.5% agarose gels with GelRed (Biotium, Hayward, CA, USA) staining. GAPDH was used as a reference. Band intensities were analyzed using Quantity One 1-D analysis software, v 4.6.5 (Bio-Rad Laboratories, Inc., Hercules, CA, USA).

**Table 3:** Primer sequences for RT-PCR

**Gene**	**Sequence (5′-3′)**
TNF-α	**F:** GTAGCCCACGTCGTAGCAAA
	**R:** CCCTTCTCCAGCTGGGAGAC
iNOS	**F:** GCAGAATGTGACCATCATGG
	**R:** ACAACCTTGGTGTTGAAGGC
TGF-β	**F:** GAGAGCCCTGGATACCAACTACTG
	**R:** GTGTGTCCAGGCTCCAAATGTAG
MR	**F:** GTAGTTCTATCTTCATCTTC
	**R:** AATATAAGACAGTCACATTA
GAPDH	**F:** TCCCTCAAGATTGTCAGCAA
	**R:** AGATCCACAACGGATACATT

*F* Forward primer, *R* Reverse primer.

### Microarray analysis

#### Sample preparation and labeling

Microarray experiments were performed in duplicate. For each sample, total RNA was extracted from tissue blocks (2 × 2 × 2 mm) obtained from four LPS-injected and uninjected rat brains using RNeasy mini kits (Qiagen GmbH, Hilden, Germany). Quantity and quality of RNA were assayed by UV spectrometry and RNA gel electrophoresis. RNA was labeled and hybridized to a GeneChip according to Standard Affymetrix Protocols (GeneChip Whole Transcript Sense Target Labeling Assay Manual, Version 4; Affymetrix, Santa Clara, CA). Affymetrix GeneChip Rat Gene 1.0 ST Arrays were used in this study. Each reaction involving a single GeneChip hybridization was initiated with 200 ng RNA. cDNA and cRNA were generated using a GeneChip WT cDNA Synthesis and Amplification Kit (900673, Affymetrix); cRNA cleanup was performed using a GeneChip IVT cRNA Cleanup Kit (900547, Affymetrix). After the second cDNA synthesis, cRNA was hydrolyzed by RNase H treatment, and biotin-labeled sense strand DNA fragments were generated using a GeneChip WT Terminal Labeling Kit (900671, Affymetrix).

#### Hybridization and scanning

Biotin-labeled DNA fragments (target) or controls in a hybridization cocktail were hybridized to the GeneChip array by incubating for 16 h in a GeneChip Hybridization Oven 640. Immediately following hybridization, the array was washed and stained with a streptavidin-phycoerythrin conjugate on the GeneChip Fluidics Station 450 using an automated protocol, followed by scanning on a GeneChip Scanner 3000 (7G). The GeneChip Hybridization, Wash, and Stain Kit (900720, Affymetrix) was used in this procedure.

#### Data measurement and analysis

An Affymetrix GeneChip scanner operated by GeneChip Operating Software (GCOS ver1.4; Affymetrix) was used to generate original array images. The average difference for each probe set (a measure of the relative abundance of a transcript) and signals and detection calls (i.e., present or absent) were computed using GCOS. Data were analyzed using Silicon Genetics Genespring 10.1 Software.

### Eriochrome cyanine RC staining

Myelin was visualized with Eriochrome Cyanine RC (ECRC; Sigma). Brain sections were mounted on slides and air-dried overnight at room temperature. After dehydration and rehydration in graded ethanol solutions, sections were stained with ECRC solution (0.2% in 0.5% H_2_SO_4_) for 10 min, rinsed in running tap water, placed in 1% NH_4_OH for 30 s, and rinsed in distilled water. After dehydration, sections were treated with xylene and mounted using Permount (Fisher Scientific Co., Morris Plains, NJ, USA).

### Measurement of damaged areas

Damaged areas were measured by staining every sixth brain section of the whole midbrain (AP, -4.3 to −6.5 mm from the bregma) with antibodies for specific markers of astrocytes (GFAP, S100β, EAAT1, and Kir4.1), endothelial cells (SMI 71), and neurites (MAP2). Myelin was stained with ECRC. Specific marker-negative areas in serial sections were measured on 4×-magnified images using Axiovision image-analysis software (version 4.7.2; Zeiss) and summed as shown in Additional file 3: Figure S3.

### Cell culture

Primary astrocytes were cultured from the cerebral cortices of 1-day-old Sprague–Dawley rats, as described previously [53,54]. In brief, cortices were triturated in MEM (Sigma) containing 10% FBS (HyClone, Logan, UT, USA), plated in 75 cm^2^ T-flasks (0.5 hemisphere/flask), and incubated for 2–3 weeks. Microglia were removed from flasks by mild shaking, and astrocytes were cultured in serum-free MEM for 2–3 d. Astrocytes were harvested with 0.1% trypsin, plated, and cultured in MEM containing 10% FBS before use. Purity of astrocytes (>95%) was confirmed using GFAP antibodies.

Rat blood monocytes were isolated as described previously [55]. Briefly, blood was obtained by cardiac puncture and mixed with 2.5% dextran in PBS for 1 h at RT. The plasma layer was centrifuged at 300× *g* for 12 min. To remove red blood cells (RBC), the pellet was suspended in PBS containing 0.15 M NH_4_Cl, 10 mM NaHCO_3_, and 0.1 mM EDTA, and centrifuged at 350× *g* for 6 min. This process was repeated twice. Pellets containing monocytes and lymphocytes were suspended in PBS and placed in 15-ml polystyrene conical tubes (BD Falcon, NJ, USA). An equal volume of Ficoll-Paque PLUS (GE Healthcare, Uppsala, Sweden) was carefully added to the bottom of cell-containing tubes so as to prevent mixing with PBS. After centrifugation at 450× *g* for 30 min, cells between the Ficoll and PBS layers were collected, washed with PBS, suspended in HBSS containing calcium (140 mg/l), and plated in a Petri dish for 30 min. Unattached lymphocytes were removed and adherent monocytes were collected and cultured in MEM containing 10% FBS.

### Astrocyte migration assay

Astrocyte migration was examined using a polydimethylsiloxane (PDMS) device [56] that was a gift from N. Jeon (Seoul National University). Briefly, PDMS device was comprised of two compartments. Each compartment had two reservoirs in the form of 8-mm-diameter holes, one at either end of the compartment, which served as a loading gate and medium reservoir, respectively (Figure 9A). The two holes in each compartment were connected by a main channel that was 1.5 mm wide, 100 μm high, and 7 mm in length. The two compartments were connected by 100 grooves, each 10 μm wide, 5 μm high, and 450 μm in length. Primary astrocytes (3 × 10^4^ cells/125-150 μl for each hole) were plated in one compartment, and culture media (150 μl for each hole) was added to the other compartment. The following day, the media was removed from the media-filled compartment and then monocytes (2.5 × 10^4^ cells/125-150 μl for each hole) were added to the compartment. The size of the grooves was sufficiently small that cells could not pass over to the opposite compartment during loading. The volume difference (50 μl) between compartments leads to convective flow from the higher volume side to the lower volume side. The difference in volume slowly decreased over time, but was still 10 μl at 7 d. Phase contrast images were taken every day and calcein AM-labeled fluorescence images were taken at 7 d using an AxioVision fluorescence light microscope (Zeiss Axiovert 200 M).

### Statistical analysis

All values are expressed as means ± SEMs. The statistical significance of differences between mean values was assessed by Student’s *t*-test using the Statistical Package for Social Sciences 8.0 (SPSS Inc., Chicago, IL, USA).

## Competing interests

The authors declare that they have no competing interest.

## Authors’ contributions

HJ designed the study and performed the bulk of the experiments and analyzed the data, and wrote the manuscript. KJ performed the experiments and analyzed the data, and supported to prepare manuscript. JK assisted with the animal experiments. IJ provided the materials and equipments in experiments. EJ supervised the design of study and coordination, analyzed the data, and wrote the manuscript. All authors have read and approved the final version of this manuscript.

## Supplementary Material

Additional file 1: Figure S1 Nissl staining showed death of neurons and glia in LPS-injected SN. (**A**) Brain sections (30 μm) prepared at 1 d after PBS- and LPS-injection were stained with Nissi. (**B**) Higher magnification images of boxed areas in (**A**) showed neurons (black arrow heads) and qua (white arrows) in the contralateral (Contra) and PBS-injected (PBS) SNpc and SNr. In LPSinjected SNpc, neurons and glia disappeared GFAP levels increased in all over the midbrain in PBS-injected brain (PBS). but disappeared in LPS-injected brain (LPS). (**C**) Higher magnification images of boxed areas in (**B**) showed infiltration of polymorphonuclei-containing neutrophils (white arrow heads) in LPS-injected brain (LPS) as shown previously (Ji et al., GLIA. 2007. 55:1577). Morphology of neutrophil’s nuclei are obviously different from that of glia (white arrows) and neurons (black arrow heads) in contralateral side and PBS-injected brain. Asterisks (*) indicate injection sites. Scale bars, 1 mm (upper panels in **A** and **B**), 50 μm (lower panels in **A**). Click here for file

Additional file 2: Figure S2 TUNEL^+^ signals were detected in lba-V round cells in LPS-injected brain. Brain sections (30 μm) were prepared at 3 d after LPS-injection, and stained with ba-i and TUNEL. TUNEL signals were detected in some of lba-1’ round cells (arrow heads). Scale bar, 20 μm. Click here for file

Additional file 3: Figure S3 Measurement of GFAP astrocyte-damaged areas. (**A**, **B**) Brain sections (30 μm) were prepared at the indicated times after PBS- and LPS-injection, and stained with GFAP antibodies, (**A**) GFAP astrocytes were found in all over the mid brain, but densities of astrocytes differ depending on regions: low in red nucleus (RN) and SNpc. but high in SNr (Intact). GFAP levels increased in all over the midbrain in PBS-injected brain (PBS). but disappeared in LPS injected brain (LPS). GFAP^**+**^ astrocytes were still detectable in the surrounding area of damage core (medial geniculate, MG). Lower panels are higher magnification images of boxed areas in upper panels. Asterisks (*) indicate injection sites Contralateral (Contra) sides of LPS-injected animals and PBS-injected animals were used as controls (**B**) GFAP-negative areas (areas within dotted lines) were measured in every sixth brain section of the whole mid brain and summed Scale bar. 1 mm (upper panels in **A** and **B**), 50 μm (lower panels in **A**). Click here for file
