# Supplementary figures and images for: Repair of astrocytes, blood vessels, and myelin in the injured brain: possible roles of blood monocytes
Source: Mol Brain. 2013 Jun 10;6:28. doi: 10.1186/1756-6606-6-28 (PMC3684510; doi:10.1186/1756-6606-6-28)

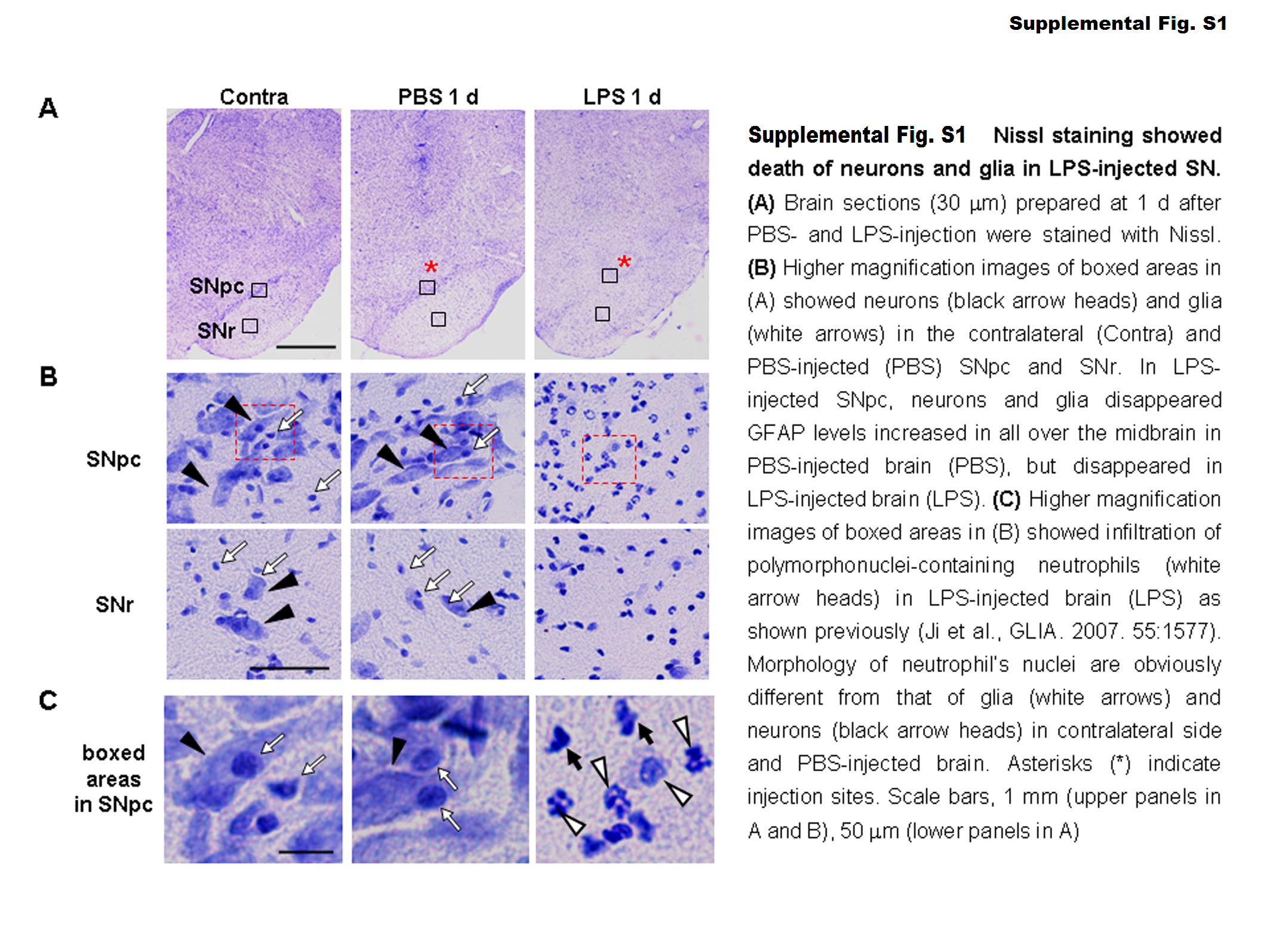

Supplement: Additional file 1: Figure S1 — Nissl staining showed death of neurons and glia in LPS-injected SN. (A) Brain sections (30 μm) prepared at 1 d after PBS- and LPS-injection were stained with Nissi. (B) Higher magnification images of boxed areas in (A) showed neurons (black arrow heads) and qua (white arrows) in the contralateral (Contra) and PBS-injected (PBS) SNpc and SNr. In LPSinjected SNpc, neurons and glia disappeared GFAP levels increased in all over the midbrain in PBS-injected brain (PBS). but disappeared in LPS-injected brain (LPS). (C) Higher magnification images of boxed areas in (B) showed infiltration of polymorphonuclei-containing neutrophils (white arrow heads) in LPS-injected brain (LPS) as shown previously (Ji et al., GLIA. 2007. 55:1577). Morphology of neutrophil’s nuclei are obviously different from that of glia (white arrows) and neurons (black arrow heads) in contralateral side and PBS-injected brain. Asterisks (*) indicate injection sites. Scale bars, 1 mm (upper panels in A and B), 50 μm (lower panels in A). [file 1756-6606-6-28-S1.tiff]

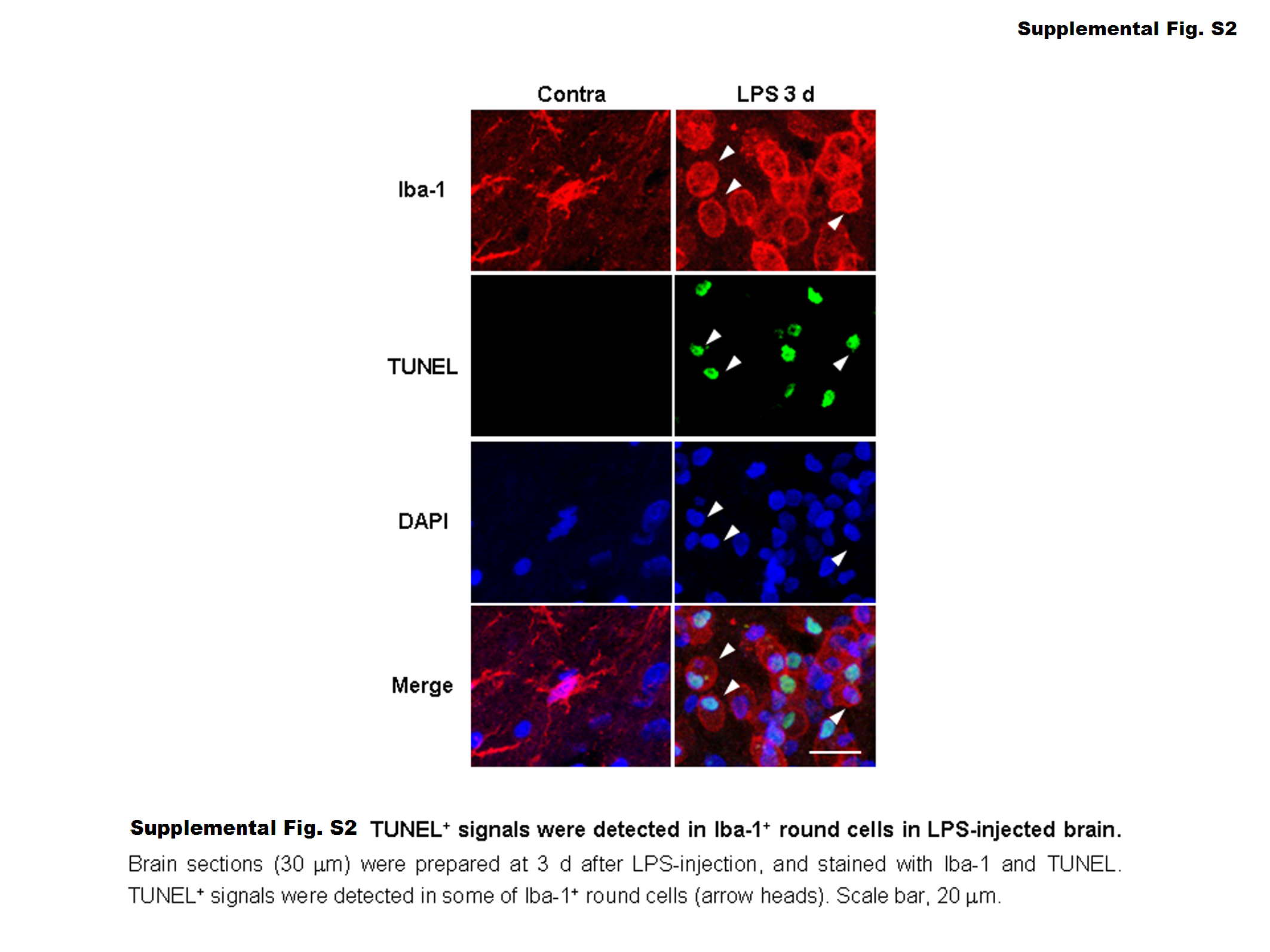

Supplement: Additional file 2: Figure S2 — TUNEL+ signals were detected in lba-V round cells in LPS-injected brain. Brain sections (30 μm) were prepared at 3 d after LPS-injection, and stained with ba-i and TUNEL. TUNEL signals were detected in some of lba-1’ round cells (arrow heads). Scale bar, 20 μm. [file 1756-6606-6-28-S2.tiff]

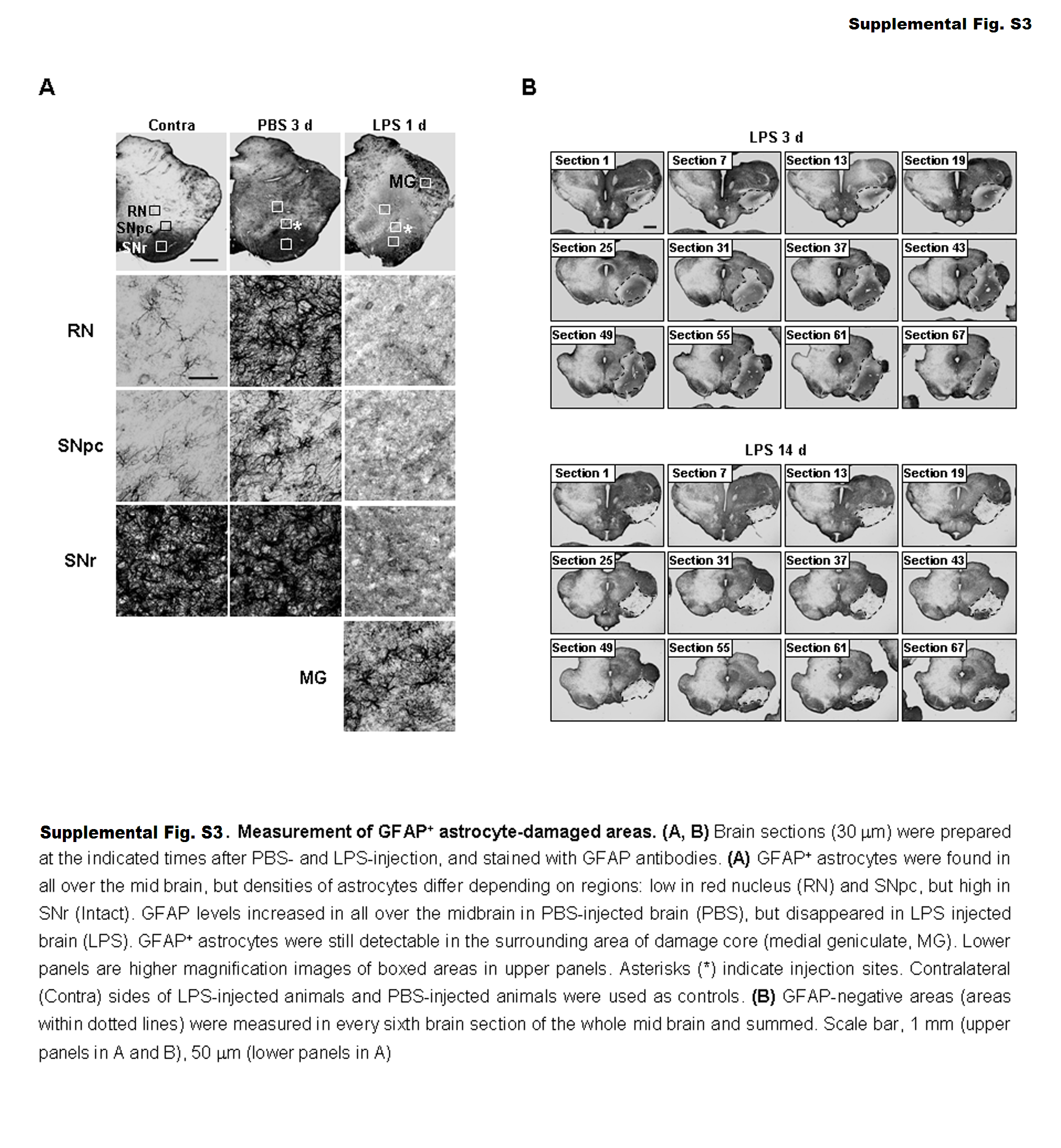

Supplement: Additional file 3: Figure S3 — Measurement of GFAP astrocyte-damaged areas. (A, B) Brain sections (30 μm) were prepared at the indicated times after PBS- and LPS-injection, and stained with GFAP antibodies, (A) GFAP astrocytes were found in all over the mid brain, but densities of astrocytes differ depending on regions: low in red nucleus (RN) and SNpc. but high in SNr (Intact). GFAP levels increased in all over the midbrain in PBS-injected brain (PBS). but disappeared in LPS injected brain (LPS). GFAP+ astrocytes were still detectable in the surrounding area of damage core (medial geniculate, MG). Lower panels are higher magnification images of boxed areas in upper panels. Asterisks (*) indicate injection sites Contralateral (Contra) sides of LPS-injected animals and PBS-injected animals were used as controls (B) GFAP-negative areas (areas within dotted lines) were measured in every sixth brain section of the whole mid brain and summed Scale bar. 1 mm (upper panels in A and B), 50 μm (lower panels in A). [file 1756-6606-6-28-S3.tiff]
